# KilR of *E. coli* Rac prophage is a dual inhibitor of bacterial cell division and elongation machineries

**DOI:** 10.1128/msphere.01029-24

**Published:** 2025-08-08

**Authors:** Anusha Marepalli, Muruganandam Nandhakumar, Sutharsan Govindarajan

**Affiliations:** 1 Department of Biological Sciences, SRM University AP 523436, Amaravati, Andhra Pradesh, India; University of Wyoming, Laramie, Wyoming, USA

**Keywords:** cell biology, bacteriophage genetics, cytoskeleton, FtsZ

## Abstract

Bacterial cryptic prophages not only encode genes that reduce the viability of the host upon induction but also contribute to host survival during stressful conditions. Rac is a cryptic prophage of *Escherichia coli*, and it encodes a toxic protein KilR, which causes morphological defects to the host. However, the mechanistic basis of its action is not well understood. In this study, we provide evidence that KilR is a dual inhibitor that affects cell division and cytoskeletal organization. We show that KilR expression is highly toxic, as demonstrated previously, and its predicted C-terminal unstructured region plays a crucial role in its function via a length-dependent manner. Low levels of KilR expression lead to cell filamentation and disruption of Z-rings, while high levels result in rod-shaped defects and mislocalization of the MreB cytoskeletal protein. Using fluorescent fusions, we observed that KilR is diffusively localized in the cytoplasm. When MreBCD proteins are overexpressed, KilR co-localizes with them, forming membrane-associated filaments, indicating a physical association. However, overexpressed MreBCD proteins do not alleviate the KilR-associated growth defect, unlike FtsZ. Finally, we present evidence that chromosomal KilR contributes to the co-inhibition of FtsZ and MreB localization in response to oxidative stress. Our data indicate that KilR inhibits MreB-associated cytoskeletal system, in addition to its effect on FtsZ-associated cell division system. We propose that the dual inhibition activity of KilR contributes to its high level of toxicity and to its function in SOS-independent DNA damage tolerance during oxidative stress.

**IMPORTANCE:**

KilR is a Rac cryptic prophage-encoded toxic protein, which contributes to host survival during oxidative stress conditions. It is known to inhibit cell division by targeting the tubulin homolog, FtsZ. In this study, we show that KilR affects FtsZ-mediated cell division and MreB-mediated cell elongation. The simultaneous inhibition of cell division and cell elongation is known to be crucial for bacterial survival during stress conditions like oxidative stress. Our study identifies KilR as a cell division and cell elongation inhibitor, offering insights into how bacterial-phage coevolution drives the emergence of cryptic prophage elements, with specific genes enhancing bacterial fitness.

## INTRODUCTION

Bacterial genomes harbor several cryptic prophages, which, unlike prophages, are no longer capable of producing infectious phage particles. These cryptic prophages persist in the bacterial genome as DNA fossil remnants for millions of years and provide selective advantages to the bacterial host. For example, several cryptic prophages encode genes for toxins, virulence factors, or antibiotic resistance, and they contribute to the bacterial survival under various stress conditions like oxidative damage, exposure to antibiotics, and during biofilm formation (1, 2).

*Escherichia coli* K-12 harbors nine cryptic prophages, constituting nearly 3.6% of its genome. Rac, the first prophage discovered in *E. coli* (2–4), is a phage fossil that was acquired over 4.5 million years ago. Rac is a defective lambdoid prophage measuring 23 kb in length and encompassing 29 genes. It is specifically integrated at the *ttcA* gene responsible for encoding tRNA-thioltransferase, TtcA. Rac can undergo excision from the *ttcA* locus during normal growth conditions, but this excision is induced under specific circumstances, such as during biofilm formation. Upon Rac excision, an altered TtcA protein (TtcA′) is formed, resulting in growth defect in the presence of antibiotics like carbenicillin (5, 6). In addition, Rac also contributes to various other phenotypes, including biofilm formation, resistance to nalidixic acid, oxidative stress tolerance, etc. (2, 5, 6), highlighting its importance as a cryptic mobile genetic element supporting bacterial survival.

KilR is a toxic protein encoded by the Rac prophage (7, 8). Under normal conditions, expression of most of the genes of the Rac prophage, including *kilR*, is tightly repressed (9, 10). However, in response to exposure to nalidixic acid or oxidative stress, KilR transcription is transiently induced, which contributes to stress survival. Many of the Rac-associated advantageous phenotypes are attributed to KilR (2, 11).

The role of KilR in bacterial survival during oxidative stress has been well investigated. OxyS sRNA is induced in response to oxidative stress (12). OxyS directly downregulates NusG, a transcription termination factor, through sRNA-mRNA interaction. NusG, together with Rho, contributes to the repression of KilR. In the presence of OxyS, the reduced expression of NusG, in turn, indirectly leads to the activation of KilR (9, 11). The transiently activated KilR inhibits FtsZ, the major cell division protein, causing temporary growth arrest in an SOS-independent manner. This arrest provides the necessary time for facilitating DNA damage repair and enabling cellular recovery (11). In addition to promoting cell division inhibition through KilR, OxyS was recently shown to inhibit cell elongation through the repression of mepS mRNA, which encodes peptidoglycan endopeptidase (13). Thus, OxyS-mediated co-inhibition of the two interlinked pathways, namely, cell division and cell elongation, has been shown to be important for cell survival and recovery from oxidative stress-induced DNA damages.

Several studies have documented that KilR expression leads to cell filamentation, indicating the inhibition of cell division (7, 8, 11). Importantly, increased expression of FtsZ, the central cell division protein, completely suppresses KilR toxicity and associated cell division defects, suggesting an association between KilR and the cell division process (7, 11). Intriguingly, it has also been observed that KilR expression leads to other morphological defects, such as the formation of lemon-shaped cells, which are hallmarks of cell elongation defects (7). Inhibition of FtsZ cannot explain cell elongation defects, indicating that KilR inhibits additional targets that mediate cell elongation. In this study, we provide evidence that KilR is a phage-encoded toxic protein, which in addition to inhibiting cell division, can also inhibit cell elongation by targeting the rod shape determining the MreBCD cytoskeletal system.

## RESULTS AND DISCUSSION

### KilR-mediated toxicity is associated with cell filamentation and rounding

**Fig 1:**
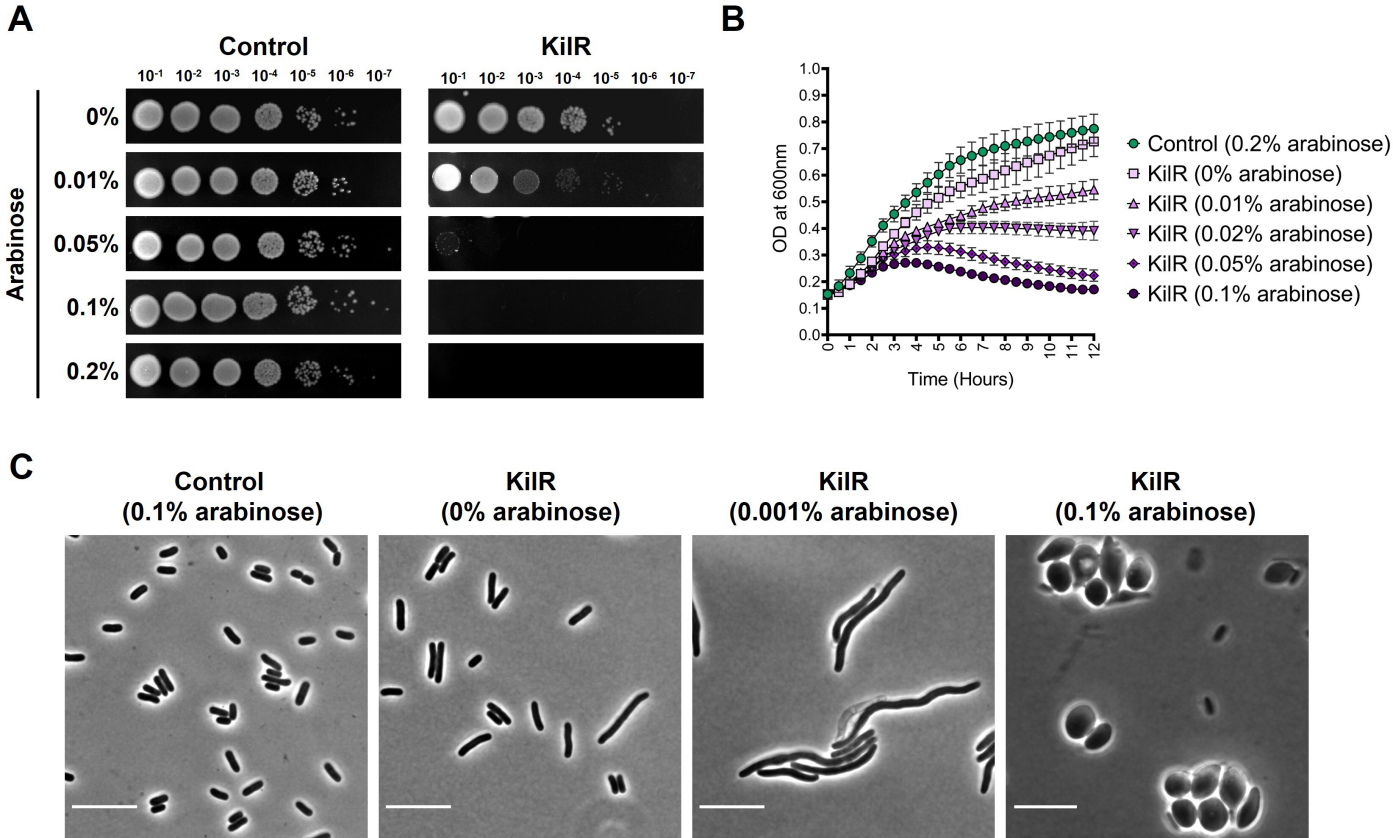
Growth inhibition and morphological effects of KilR expression. (**A**) Wild-type cells (MG1655) transformed with pBAD-mCherry (control) or pBAD-KilR were spotted in serial dilutions on LB plates without or with the inducer arabinose at varying concentrations and incubated overnight at 37°C. (**B**) Growth curves of cells expressing KilR in the presence of different concentrations of arabinose or the mCherry control (induced with 0.2% arabinose). (**C**) Phase contrast images of wild-type cells expressing mCherry control or KilR at various concentrations of inducer arabinose. Scale bar corresponds to 10 µm.

In order to investigate the impact of KilR on *E. coli* growth, we constructed a plasmid expressing KilR under an arabinose-inducible promoter. We assessed the growth of *E. coli* expressing KilR or a control protein, mCherry, by spotting serial dilutions of overnight cultures onto LB agar plates with or without varying concentrations of arabinose. The results summarized in Fig. 1A indicate that KilR displayed a concentration-dependent inhibition of cell growth. At a lower arabinose concentration (0.01%), a slight defect in cell viability was observed. However, at higher concentrations, growth was completely inhibited, consistent with previous observations of KilR’s toxicity (7). KilR-mediated growth arrest was also evident in liquid growth assays in an inducer concentration-dependent manner (Fig. 1B).

Next, we investigated the morphological effects associated with the KilR expression. The expression of KilR at a low concentration of the inducer arabinose resulted in the formation of severely elongated cells, indicating inhibition of cell division. In contrast, at higher inducer concentrations, the KilR expression predominantly led to the formation of lemon-shaped or spherical cells, a hallmark of defects in cell elongation. In the absence of any inducer, cells appeared slightly longer compared to cells expressing a control mCherry protein, which appeared normal (Fig. 1C). While similar morphological defects were reported for KilR in one study (7), discrepancies exist in other reports in which KilR expression only results in cell filamentation (8, 11). To validate our findings, we assessed the morphological defects associated with the KilR expression using a previously reported plasmid, pSA97 (11). Consistent with our observations, KilR expressed from pSA97 induced cell filamentation at low concentrations of the inducer and formed lemon- or round-shaped cells at high concentrations of inducer (see Fig. S1 at https://doi.org/10.5281/zenodo.15803833).

The phenotypes associated with the KilR overexpression resemble those induced by other proteins targeting both cell division and cell elongation. This includes CptA, a host-encoded toxin part of the CptA-CptB toxin-antitoxin system (14), and CbtA, a toxin encoded in the CP4-44 prophage CbtA-CbeA toxin-antitoxin system (15, 16). Despite lacking homology with KilR, these toxin proteins exhibit comparable morphological effects. Notably, they are part of a toxin-antitoxin system and known to play a crucial role in oxidative stress tolerance (17). However, in contrast, it remains uncertain whether KilR is part of a toxin-antitoxin system. Nevertheless, the results presented above suggest that KilR serves as a dual inhibitor capable of affecting both cell division and cell elongation in *E. coli*.

### Effect of KilR is distinct compared to λ kil

**Fig 2:**
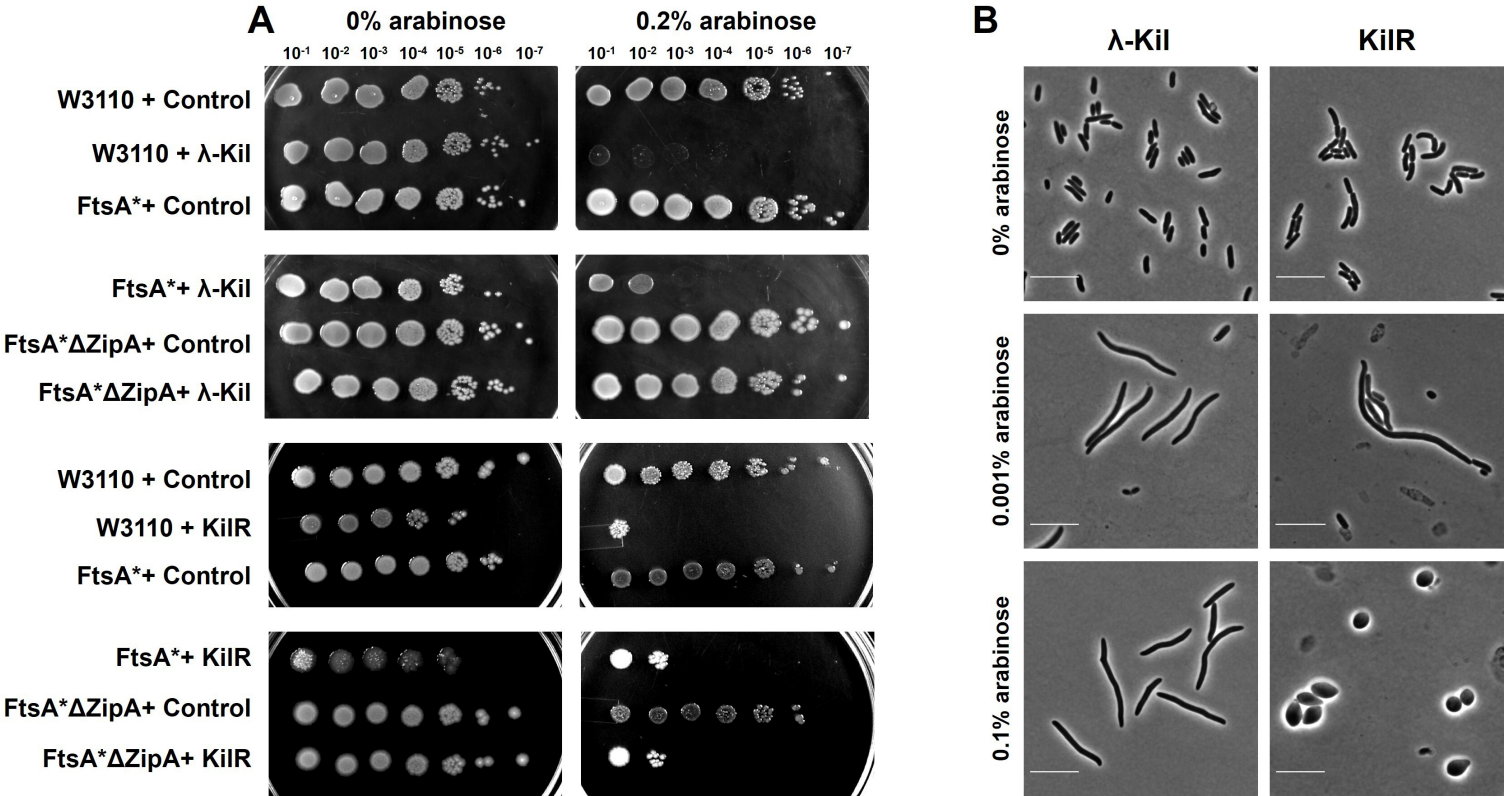
Comparison of λ Kil and Rac KilR. (**A**) Serial dilutions of the specified strains transformed with plasmids expressing control mCherry, λ Kil, or KilR were cultured under conditions where λ Kil expression is induced (at 42°C) as previously described in reference 19. Plasmids were either left uninduced or induced with 0.2% arabinose. (**B**) Phase contrast images of wild-type cells (W3110) expressing λ Kil (left panel) or KilR (right panel) with different concentrations of inducer arabinose. Scale bar corresponds to 10 µm.

Similar to the KilR of the Rac prophage, the λ phage also encodes a toxic Kil peptide that affects cell growth (18). Although Rac KilR and λ Kil are not related, both proteins are known to target FtsZ. In the case of λ Kil, FtsZ inhibition requires the presence of another cell division protein ZipA (19, 20). FtsA* (R286W) is a gain-of-function mutation that causes an altered FtsA-FtsZ interaction that can bypass the requirement for several cell division proteins, including ZipA (21). Interestingly, a previous study has shown that in the mutant background of ftsA*ΔzipA, the toxicity of λ Kil is completely abolished (19). Since KilR targets FtsZ similar to λ Kil, we tested whether ftsA*ΔzipA background could rescue KilR toxicity. To investigate this, we tested the toxicity of KilR in ftsA* and ftsA*ΔzipA strains, comparing it with λ Kil. The results presented in Fig. 2A show that the expression of λ Kil in ftsA* did not confer resistance to cells. Consistent with a previous report (19), double-mutant ftsA*ΔzipA cells exhibited complete resistance to the λ Kil expression. However, in both ftsA* and ftsA*ΔzipA strains, the KilR expression was toxic to comparable levels (Fig. 2A), suggesting that KilR mediates its function distinctly from λ Kil. To further evaluate this observation, we compared the effects of λ Kil and KilR on *E. coli* cell morphology. In the presence of low concentration of the inducer, both λ Kil and KilR caused cell filamentation. However, in the presence of a high inducer concentration, cells carrying λ Kil exhibited increased filamentation, whereas cells expressing KilR became round (Fig. 2B). Thus, although Rac KilR and λ Kil target FtsZ, their additional targets are distinct. For λ Kil, ZipA is the additional target, but for KilR, the additional target is unidentified. The formation of lemon-shaped or spherical cells is a characteristic feature of cell elongation defects. Such phenotypes are observed in cell elongation-defective mutants disrupted for the MreBCD cytoskeletal system or its associated proteins, including RodZ or RodA (22). Since increased expression of KilR caused these morphological phenotypes, it suggests the possibility of cell elongation-associated cytoskeletal components as additional targets.

### C-terminal region of KilR is critical for its toxicity and dual morphological effects

**Fig 3:**
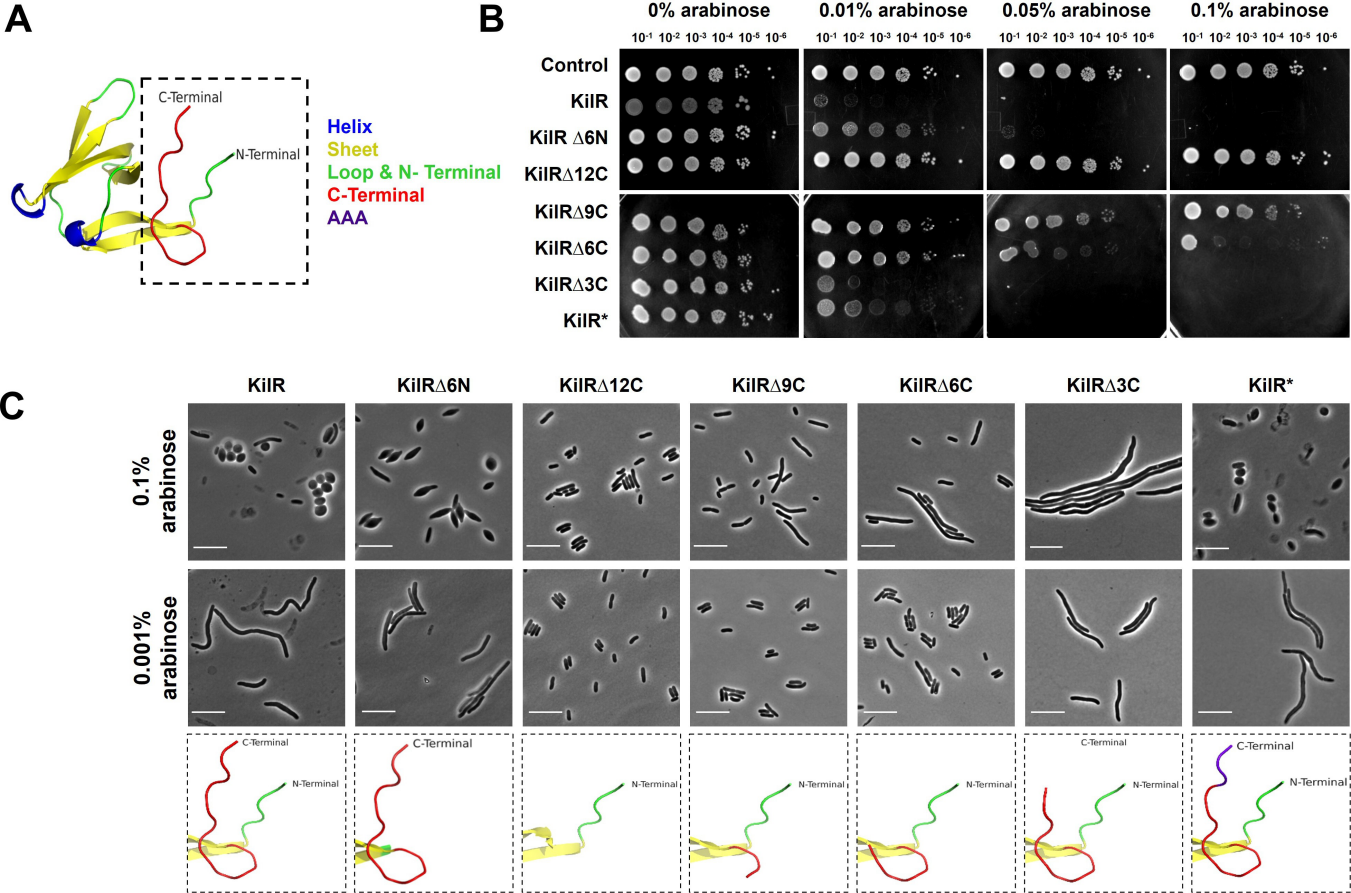
C-terminal unstructured region of KilR is critical for its function. (**A**) AlphaFold3 predicted structure model of KilR. (**B**) Serial dilutions of the wild type (MG1655) transformed with plasmids expressing control mCherry, KilR, KilR∆6N, KilR∆12C, KilR∆9C, KilR∆6C, KilR∆3C, and KilR* (3 Ala variant) without or with the inducer arabinose at varying concentrations and incubated overnight at 37°C. (**C**) Phase contrast images of wild-type cells expressing KilR, or specified KilR variants. Scale bar corresponds to 10 µm. Models depicting the N- and C-terminal regions are shown below the respective KilR versions.

To further understand the nature of KilR, we predicted its structure using AlphaFold3 (23). We obtained a structure with a predicted local distance difference test (pLDDT) of 91.3% suggesting it is a high-confidence predicted structure. The predicted structure of KilR showed that the central region of the protein is largely composed of beta sheets, but two unstructured regions corresponding to seven and 12 amino acids were present in the N- and C-terminals, respectively (Fig. 3A). Short unstructured regions at the N- and C-termini of KilR were also predicted using IUpred (24), a tool for identifying disordered protein regions using the short disorder option (see Fig. S2 at https://doi.org/10.5281/zenodo.15803833).

To investigate the importance of these unstructured regions in KilR‘s function, we generated KilR mutants lacking the N- (KilR∆6N) and C-terminal (KilR∆12C) unstructured regions and assessed their impact on toxicity and morphological effects. The results presented in Fig. 3B show that the deletion of the N-terminal unstructured region is largely similar to the wild type with slight differences in toxicity at low concentration but not at high concentration. In contrast, the deletion of the C-terminal unstructured region completely abrogated the KilR toxicity. Morphological analysis supports the toxicity assay, showing that cells expressing KilR∆6N appeared lemon-shaped, while those expressing KilR∆12C appeared completely normal (Fig. 3C).

To further investigate the critical regions within the C-terminal unstructured region, we generated a series of truncations by deleting the last nine amino acids (KilRΔ9C), six amino acids (KilRΔ6C), and three amino acids (KilRΔ3C) and assessed their effects. KilRΔ9C and KilRΔ6C showed a gradual reduction in cell viability, with KilRΔ6C still causing a slight filamentation (Fig. 3B and C). Interestingly, KilRΔ3C, which lacks only the last three amino acids, retained its toxicity but lost the ability to induce cell rounding defects, instead leading to the formation of filamented cells (Fig. 3B and C).

Next, we wanted to know whether the specific nature of the last three amino acids of KilR (Glu-Ser-Trp) is critical for the observed effects. To check this, we created a KilR variant, termed KilR*, in which the last three amino acids were replaced with three alanines. Remarkably, KilR* regained the ability to inhibit cell elongation and exhibited toxicity and morphological defects similar to the wild-type KilR (Fig. 3B and C; also see Fig. S3 at https://doi.org/10.5281/zenodo.15803833). To check whether this is due to differences in expression levels, we tagged the proteins with mCherry at the N-terminus and analyzed them. Toxicity analysis showed that mCherry-tagged proteins exhibited slight differences compared to untagged versions, potentially due to the presence of the large tag. However, microscopy analysis showed that the mCherry-tagged proteins displayed morphological features similar to the untagged proteins (see Fig. S4 at https://doi.org/10.5281/zenodo.15803833). Western blot analysis of the mCherry-tagged wild type, C-terminal truncated proteins, and KilR* revealed that all proteins were expressed as full-length forms at comparable levels, although fragmented variants were also detected (see Fig. S5 at https://doi.org/10.5281/zenodo.15803833). These results suggest that the observed differences are not due to a lack of protein expression, which can sometimes result from C-terminal mutations (25).

Collectively, these data support the following conclusions: (i) the C-terminal unstructured region of KilR is crucial for mediating its toxicity and represents the active region involved in inhibiting cell division and cell elongation; (ii) the gradual reduction of toxicity observed with progressive truncation of the C-terminal unstructured region suggests that its length contributes to the strength of KilR toxicity; and (iii) the last three amino acids, independent of their identity, potentiate KilR’s ability to inhibit cell elongation. Additionally, the deletion of the last three amino acids allowed us to uncouple the cell division and cell elongation inhibition caused by KilR without compromising its toxicity. While these results provide insights into the functioning of KilR, a detailed understanding of its mechanism of action will require a structural analysis of KilR, particularly the C-terminal unstructured region, in the presence and absence of its binding partners.

### KilR perturbs Z-ring formation and MreB localization

Having observed that the KilR expression causes cell division as well as cell elongation defects, we next checked the effect of KilR and its truncated variants on the subcellular organization of FtsZ and MreB, which mediate cell division and elongation, respectively (26–29). To answer this question, we used a strain that expressed ZapA-GFP and MreB-RFP^SW^ from the native locus (30–32). ZapA is an early cell division protein that co-localizes with FtsZ, and a C-terminal GFP-tagged, natively expressed version of ZapA can be reliably used as a marker for FtsZ localization (31, 33, 34). MreB-RFP^SW^ is a sandwich fusion protein in which the fluorescent RFP is inserted between helices 6 and 7 of MreB. Chromosomally expressed MreB-RFP^SW^ serves as a reliable marker for cytoskeletal localization (30).

**Fig 4:**
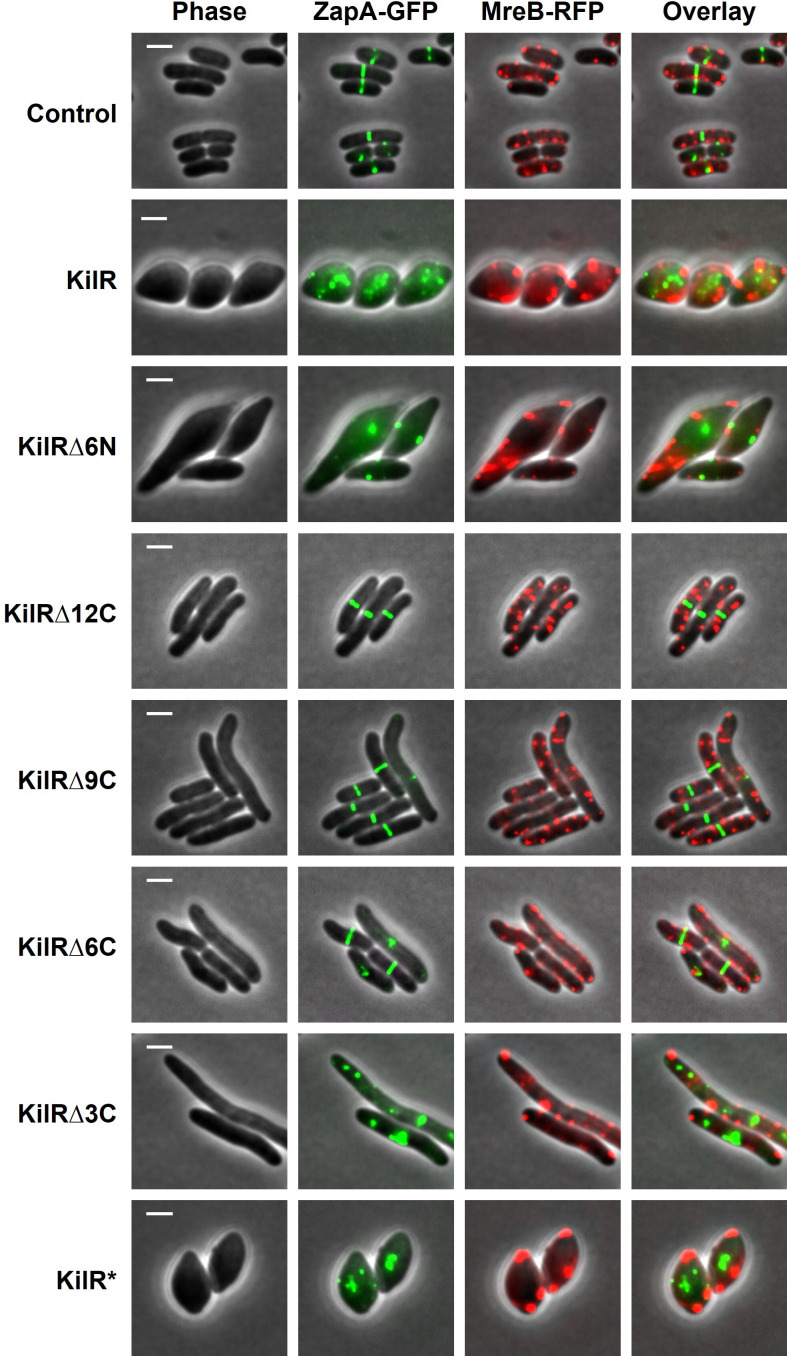
KilR overexpression affects the localization of FtsZ and MreB. Fluorescence microscopy images of cells expressing ZapA-GFP and MreB-RFP^SW^ (in MG1655 background) and transformed with plasmids expressing control (GST), wild-type KilR, its truncated versions (KilR∆6N, KilR∆12C, KilR∆9C, KilR∆6C, or KilR∆3C), or KilR* (3 Ala variant) induced with 0.1% arabinose. The GFP and mCherry fusion proteins were observed by fluorescence microscopy, and cells were observed with phase contrast microscopy. Shown are phase contrast (gray), GFP (green), and mCherry (red) fluorescence signals. Fluorescent signals are overlaid on phase contrast images. Scale bar corresponds to 2 µm.

The results presented in Fig. 4 reveal that KilR overexpression caused significant perturbations in the localization patterns of both ZapA-GFP and MreB-RFP^SW^. Specifically, ZapA-GFP exhibited a spotty appearance, losing its characteristic Z-ring formation, while MreB-RFP^SW^ formed aberrant, mislocalized clusters. In contrast, control cells displayed clear mid-cell localization of ZapA-GFP and membrane-associated spotty localization of MreB-RFP^SW^, as previously observed (32).

Cells expressing KilR∆6N showed disruptions in ZapA-GFP and MreB-RFP^SW^ localization, similar to wild-type KilR. Conversely, in cells expressing KilR∆12C, both ZapA-GFP and MreB-RFP^SW^ localizations appeared completely normal, indicating that the absence of the last 12 amino acids abolished KilR’s inhibitory effects on Z-ring formation and MreB localization. Cells expressing KilR∆9C and KilR∆6C exhibited partial effects: ZapA-GFP formed Z-rings in most cells, except for those displaying slight elongation, and MreB-RFP^SW^ localization appeared largely normal. In contrast, the expression of KilR∆3C significantly disrupted ZapA-GFP localization but resulted in a mixed pattern of MreB-RFP^SW^ localization, with aberrant clusters alongside normal membrane-associated small spotty clusters. This observation explains the maintenance of rod-shaped morphology during KilR∆3C overexpression. Finally, cells expressing KilR* disrupted both ZapA-GFP and MreB-RFP^SW^ localization patterns, mirroring the effects of wild-type KilR (Fig. 4). Unlike KilR, the expression of λ Kil induces cell filamentation without disrupting MreB localization (see Fig. S6 at https://doi.org/10.5281/zenodo.15803833). Taken together, these findings suggest that KilR, via distinct regions of its C-terminal region, modulates the subcellular organization of FtsZ and MreB, thereby influencing cell division and elongation, respectively.

### Overexpression of MreBCD recruits KilR but fails to counteract KilR-mediated cell death

**Fig 5:**
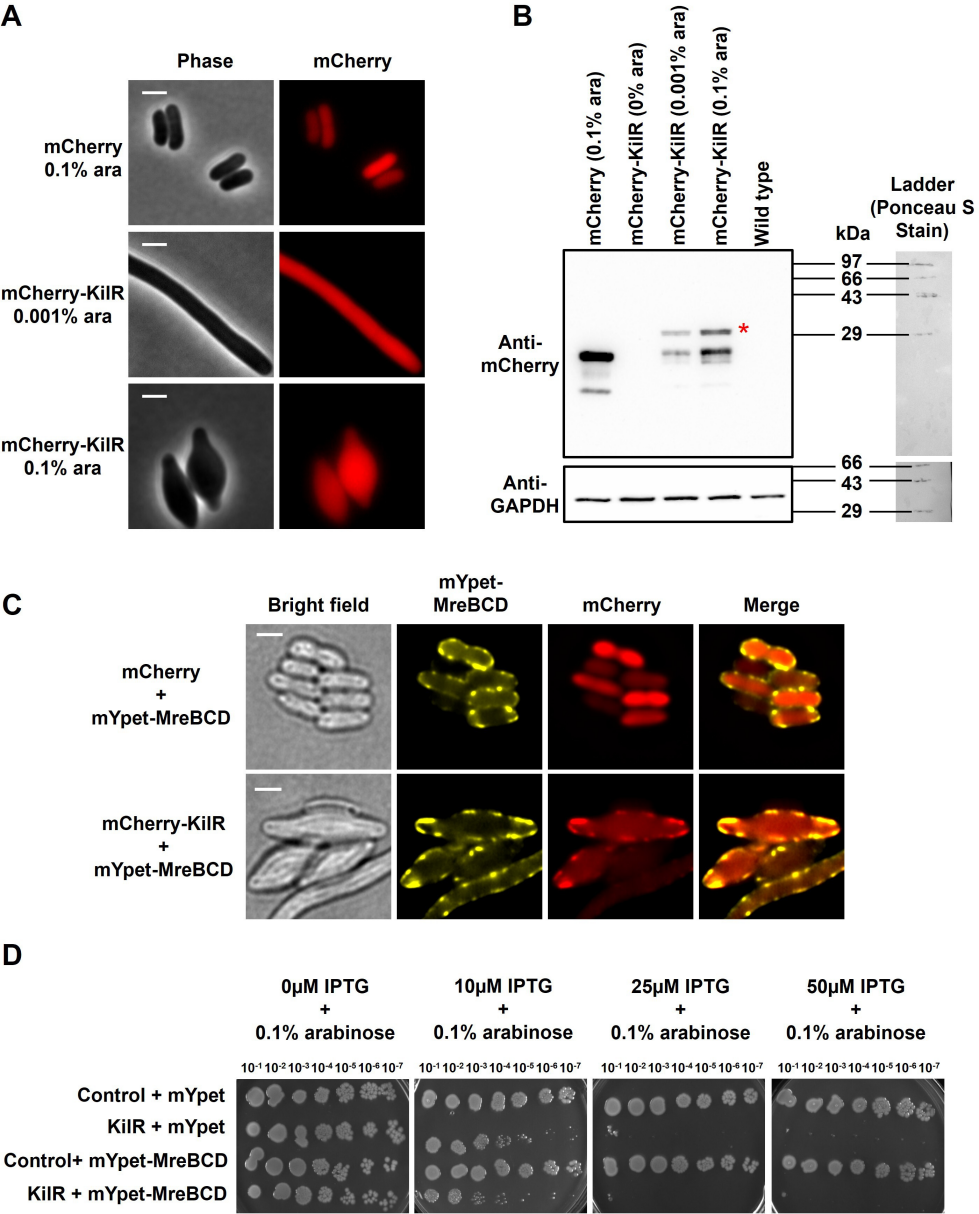
Effect of MreBCD overexpression on KilR localization and function. (**A**) Fluorescence microscopy images of wild-type cells (MG1655) expressing mCherry or mCherry-KilR with indicated arabinose concentration. mCherry was observed by fluorescence microscopy, and cells were observed with phase contrast imaging. Shown are phase contrast (gray) and mCherry (red) fluorescence signals. (**B**) Western blot analysis shows the protein expression in control cells (BL21) and in cells expressing either mCherry or mCherry-KilR under uninduced and arabinose-induced conditions at specified concentrations (top panel). The band corresponding to full-length mCherry-KilR (33.4 kDa) is marked with a red asterisk. Western blotting against GAPDH (36 kDa) was performed as the housekeeping control (lower panel). The protein ladder was visualized using Ponceau S staining of the membrane. (**C**) Fluorescence microscopy images of wild-type cells (MG1655) containing mYpet-MreBCD and co-expressing mCherry or mCherry-KilR and induced with 0.4% arabinose. The mYpet and mCherry fusion proteins were observed by fluorescence microscopy, and cells were observed with phase contrast microscopy. Shown are phase contrast (gray) and mYpet (yellow) and mCherry (red) fluorescence signals. Scale bar corresponds to 2 µm. (**D**) Serial dilutions of the wild type transformed with specified plasmids and grown without or with the inducer arabinose at varying concentrations and incubated overnight at 37°C.

To investigate the cellular distribution of KilR and its interactions with cytoskeletal components, we engineered a fluorescently tagged version of KilR by fusing mCherry to its N-terminus. The mCherry-KilR fusion protein showed a slight change in toxicity (see Fig. S4A at https://doi.org/10.5281/zenodo.15803833), whereas the morphological defects were similar to those caused by untagged KilR (Fig. 5A). Fluorescence microscopy showed that mCherry-tagged wild-type KilR and its variants exhibited diffuse cytoplasmic localization, similar to the control protein mCherry (Fig. 5A; also see Fig. S4B at https://doi.org/10.5281/zenodo.15803833). Western blot analysis confirmed the expression of full-length mCherry-KilR, although slight degradation products appeared. Furthermore, western blot analysis indicates that overall KilR expression is lower under conditions of cell filamentation, whereas it is relatively higher during cell rounding (Fig. 5B).

The MreBCD proteins form a membrane-associated complex that co-localizes with interacting proteins, such as RodZ and MbiA (35–37). We hypothesized that KilR might associate with the MreBCD complex, influencing its subcellular distribution. To test this, we co-expressed mYpet-tagged MreBCD along with either mCherry-KilR or mCherry alone and analyzed their localization patterns. As expected, mypet-MreBCD formed membrane-associated filaments when overexpressed from a plasmid, consistent with previous reports (38). Remarkably, in cells co-expressing mCherry-KilR and mYpet-MreBCD, mCherry-KilR co-localized with the membrane-associated MreBCD filaments (Fig. 5C, lower panel). In contrast, control cells expressing mCherry alone showed no co-localization with mYpet-MreBCD (Fig. 5C, upper panel). This observation indicates that KilR associates with the MreBCD complex.

Next, we assessed whether MreBCD overexpression could counteract the toxic effects of KilR. As shown in Fig. 5D and Fig. S7 at https://doi.org/10.5281/zenodo.15803833, overexpressing MreBCD did not mitigate KilR-mediated toxicity. Western blot analysis confirmed that, under our experimental conditions, MreBCD proteins were expressed at levels 2.6-fold higher than the native levels (see Fig. S8 at https://doi.org/10.5281/zenodo.15803833). Although overexpressed MreBCD could not suppress toxicity, it did reduce the cell rounding effects associated with the KilR expression, resulting in more elongated cells compared to cells expressing the control mYPet, which exhibited elongation defects during the KilR expression (see Fig. S9 at https://doi.org/10.5281/zenodo.15803833). This contrasts with the complete suppression of KilR toxicity and associated morphological defects observed during FtsZ overexpression (7, 11). Our findings suggest that KilR associates with the MreBCD cytoskeletal complex; however, increased levels of MreBCD proteins fail to inhibit KilR toxicity. The reason for this remains unclear, but it may be due to partial sequestration of KilR and continued inhibition of FtsZ, as indicated by persistent cell filamentation.

### KilR contributes to dual inhibition of FtsZ and MreB during oxidative stress

Under normal growth conditions, *kilR* is tightly repressed (9, 10), but its expression is induced during specific stress conditions, such as oxidative stress (11). Since plasmid-expressed KilR can inhibit both processes, our next investigation focused on whether a similar effect could be observed for the native chromosomal KilR through induction of oxidative stress. To explore this, we examined the localization of FtsZ-mCherry expressed from a plasmid and MreB-msfGFP^SW^ expressed from the native locus in both wild-type and *ΔkilR* mutant strains. Oxidative stress was induced by adding various concentrations of H_2_O_2_, and the effects on protein localization were assessed.

**Fig 6:**
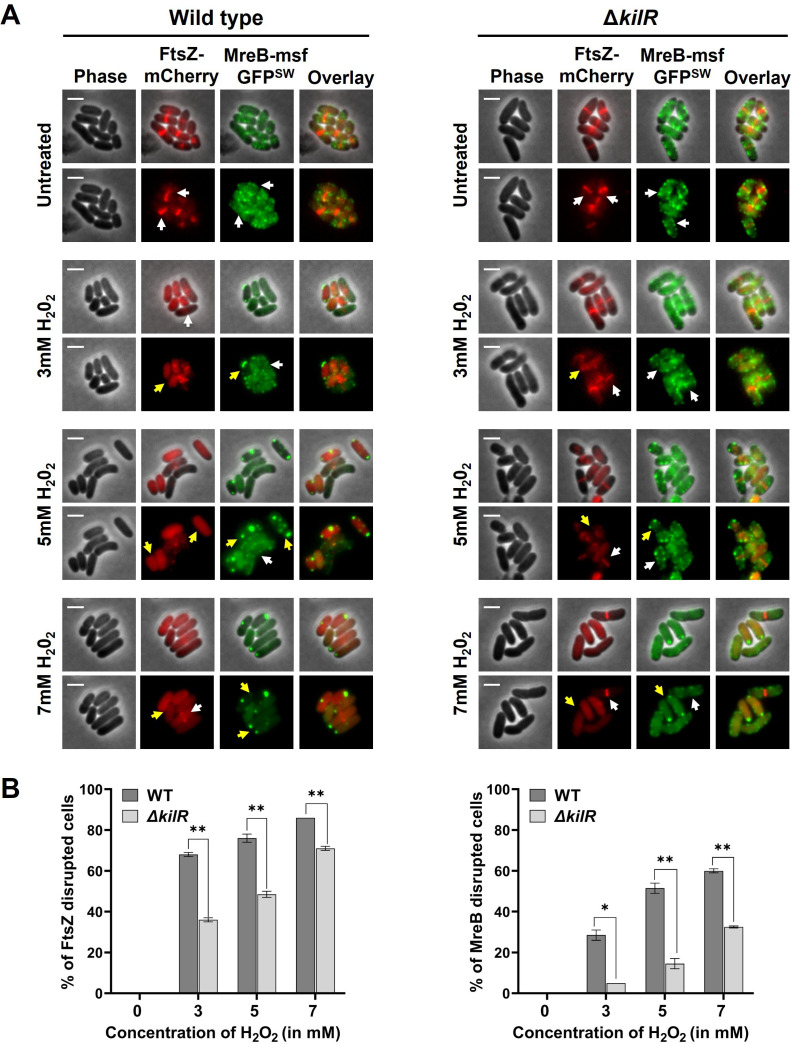
KilR affects FtsZ and MreB localization during oxidative stress. Fluorescence microscopy images of wild-type and Δkilr cells expressing FtsZ-mCherry and MreB-msfGFP^SW^ and untreated or treated with H_2_O_2_ at various concentrations. The mCherry and GFP fusion proteins were observed by fluorescence microscopy, and cells were observed with phase contrast microscopy. Shown are phase contrast (gray) and mCherry (red) and GFP (green) fluorescence images as well as overlay over phase contrast images. Cells with normal Z-rings and MreB localization are indicated with white arrows. Cells with mislocalized Z-rings and MreB are indicated with yellow arrows. Scale bar corresponds to 2 µm. (**B**) Percentage of cells with mislocalized FtsZ-mCherry (left) or MreB-msfGFP^SW^ (right) is shown. The data represent approximately 200 cells analyzed from two independent experiments. Means and standard deviations are shown. The statistical significance was calculated using unpaired *t*-test analysis (*P < 0.05, **P < 0.01).

In wild-type cells, oxidative stress caused a clear disruption of both FtsZ-mCherry and MreB-msfGFP^SW^ localizations compared to untreated cells. Mislocalization of FtsZ-mCherry was evident at even low concentrations of H₂O₂ (3 mM), whereas MreB mislocalization occurred only at higher concentrations (5 and 7 mM) (Fig. 6A; also see Fig. S10 at https://doi.org/10.5281/zenodo.15803833).

Interestingly, in the ∆*kilR* background, the mislocalization of both FtsZ-mCherry and MreB-msfGFP^SW^ was less pronounced following H_₂_O_₂_ treatment (Fig. 6A and B; also see Fig. S10 at https://doi.org/10.5281/zenodo.15803833). For FtsZ-mCherry, significant differences were observed at 3 and 5 mM H₂O₂ concentrations, whereas for MreB-msfGFP^SW^, significant differences were noted at 5 and 7 mM H₂O₂ concentrations (Fig. 6B). Overall, these findings suggest that KilR, when expressed under physiologically relevant stress conditions, such as oxidative stress, mediates the dual inhibition and mislocalization of FtsZ and MreB. The observed effect is stronger for FtsZ compared to MreB. Furthermore, under the tested conditions, we observed disruptions in the localization of FtsZ and MreB without inducing any morphological defects.

### Conclusion

The results of this study establish KilR as a phage-encoded morphogenetic inhibitor capable of dual inhibition of FtsZ-mediated cell division and MreB-mediated cytoskeletal processes. The characterization of KilR reveals that a 12-amino acid unstructured region in the C-terminus, as predicted by AlphaFold3, is important for its dual inhibitory function. However, a KilR variant lacking the last three amino acids lost its dual-targeting ability, resulting in inhibition of only the cell division process. Replacing these three amino acids with alanines restored KilR’s dual-targeting activity. These observations suggest that the length of the unstructured C-terminal region provides flexibility, potentially enabling interactions with multiple targets, while the identity of the last three amino acids is not critical. Localization studies demonstrated that KilR diffuses cytoplasmically but associates with the MreBCD complex upon its overexpression. Despite this co-localization, elevated MreBCD levels failed to counteract KilR-mediated cell death, suggesting a dominant inhibitory effect of KilR presumably due to its dual inhibitory effect on FtsZ-associated cell division and MreB-associated cytoskeletal processes. While this study provides valuable insights into the dual inhibitory function of KilR, several aspects remain to be explored in greater detail. This includes uncovering the cellular targets of KilR and understanding how these interactions modulate both FtsZ and MreB functions. Furthermore, future studies focusing on identifying and characterizing these interaction partners, along with structural analyzes of KilR in complex with its targets, will be essential for gaining a more comprehensive understanding of its inhibitory mechanisms.

The dual targeting of cell division and cytoskeletal organization has recently been proposed to contribute to bacterial survival and their ability to cope with DNA damage during oxidative stress (13). The OxyS small RNA induced in response to oxidative stress possesses antimutator properties by reducing bacterial DNA damage capacity after exposure to oxidative stress (11, 12). Previous studies have indicated that dual inhibition of cell division and the cytoskeleton is orchestrated by two distinct pathways of OxyS. In one pathway, OxyS induces KilR, which causes SOS-independent blockade of cell division (11), while in another pathway, OxyS reduces the expression of *mepS*, the gene encoding a peptidoglycan endopeptidase crucial for cell elongation (13). Our findings, revealing that KilR itself is capable of dual targeting, suggest the redundancy of this pathway and underscore its significance in oxidative stress tolerance.

## MATERIALS AND METHODS

### Bacterial strains and growth media

Strains and plasmids used in this study are listed in supplementary Table S1 at https://doi.org/10.5281/zenodo.15803833. Overnight *E. coli* cultures were grown in LB at 37 or 30°C depending on the experiment and supplemented with appropriate antibiotics. When appropriate, antibiotics were added at the following concentrations: ampicillin (100 µg/mL), kanamycin (50 µg/mL), or chloramphenicol (30 µg/mL) (Sisco Research Laboratories).

### Construction of strains and plasmids

AM001, which expresses MreB-msfGFP^SW^ in *ΔkilR* background, was constructed by P1-transduction transferring mreB′-msfGfp-′mreBcsrD(::neo) from NO50 (39) to A870 (11). Plasmids expressing mCherry, KilR, and KilR variants were constructed by Gibson assembly in the pBAD18 plasmid. mCherry was amplified from pSG30T-sfCherry-csy1(IF) (40) using the primers F-pBAD18-rbs-cherry and R-pBAD18-mcherry, and kilR was amplified from the genomic DNA of *E. coli* MG1655 K12 using F-vec-rbs-Kil and R-vec-kilR primers. C-terminal truncations of KilR were made as follows: for pBAD-KilRΔ3C, kilR was amplified with F-vec-rbs-Kil and R-KilR∆3C primers, and pBAD-KilRΔ6C, pBAD-KilRΔ9C, pBAD-KilRΔ12C, and KilR* were constructed in a similar manner. However, in all these plasmids, the forward primer for the kilR gene was the same (F-vec-rbs-Kil), and the reverse primers were R-KilR∆6C (for pBAD-KilR Δ6C), R-KilR∆9C (for pBAD-KilR Δ9C), and R-KilR∆12C (for pBAD-KilR Δ12C) R_KilR* (for KilR*). For N-terminal truncation of KilR (KilR∆6N), kilR was amplified using F-KilR∆6N and R-vec-kilR primers. For pBAD-GST, GST was amplified from pGex-6p-2 using F-pBAD18-GST and R-pBAD18-GST. The resulting amplicons were Gibson-assembled in the pBAD18 vector backbone amplified using F-pBAD18-gib and R-pBAD18 gib primers.

pBAD-mCherry-KilR fusions were constructed as follows: for pBAD mCherry-KilR, mCherry was amplified without the stop codon using F-pBAD18-rbs-cherry and R-KilR-sfCherry (-TAA) primers. KilR was amplified without a start codon using LIN-KilR Gib (-ATG) and R-vec-kilR primers. pBAD-mCherry-KilRΔ3C was constructed using kilR amplified with F-sfC-Lin-KilR and R-KilR∆3C primers, and the pBAD18 vector backbone was amplified along with mCherry using F-pBAD18-gib and R-Lin-sfC primers. pBAD-mCherry-KilRΔ6C, pBAD-mCherry-KilRΔ9C, pBAD-mCherry-KilRΔ12C, and pBAD-mCherry-KilR* were constructed in a similar manner. However, in all these plasmids, the forward primer for the kilR gene was the same (F-sfC‘-Lin-KilR), and the reverse primers were R-KilR∆6C (for pBAD-KilR Δ6C), R-KilR∆9C (for pBAD-KilR Δ9C), and R-KilR∆12C (for pBAD-KilR Δ12C). mCherry-KilR and C-terminal truncations of KilR with mCherry fusion have a ggaggcggtggagcc (G-G-G-G-A) linker sequence between them.

### Spot titer and growth curve assays

For spot titer assays, overnight cultures were serially diluted 10-fold in fresh LB medium. Subsequently, 3 µL of the serially diluted culture was spotted onto LB plates containing the appropriate antibiotic(s) and inducers. The plates were then incubated at the specified temperatures for 12–16 h. The images were captured using Bio-Rad ChemiDoc XRS+ Imaging System and processed using Fiji (ImageJ) software. For the growth curve analysis, overnight cultures were diluted to 1:1,000 and grown for 2 h in fresh LB at 37°C with the appropriate antibiotic. After 2 h, the inducer arabinose was added at specified concentrations. Then, 180 µL from each sample was aliquoted into a fresh 96-well plate and sealed with a microplate seal film, which was punctured with a sterile needle for aeration. Subsequently, the 96-well plate was loaded into a Spark multimode microplate reader, and optical density (OD) was measured at 600 nm every 15 min for a duration of 12 h. The resulting graph was plotted using GraphPad Prism.

### Expression analysis using western blotting

BL21 *E. coli* cells were transformed with plasmids expressing mCherry-tagged KilR and its truncated variants. mCherry-tagged proteins were expressed as previously described (41). For the KilR expression, BL21 was used because we noticed that it resulted in less degradation compared to MG1655, potentially due to its Lon-defective nature. For comparing the expression of mCherry-tagged wild-type with that of other variants, overnight cultures were diluted to 1:5 in LB media containing ampicillin and induced with 0.05% arabinose for 4 h. For checking the expression of wild-type KilR tagged with mCherry in different expression conditions, overnight cultures of *E. coli* cells expressing mCherry-KilR were diluted 1:5 in LB media containing ampicillin and induced with 0, 0.001, and 0.1% arabinose for 4 h. In general, after protein induction, equal volumes of samples were collected, washed, and prepared for analysis. Protein samples were separated on a 12% SDS-PAGE gel and transferred to a 0.45 µm nitrocellulose membrane (Cytiva) using the Bio-Rad Mini-PROTEAN Tetra Vertical Electrophoresis System in 1× Tris/glycine buffer. The membranes were blocked with 5% BSA in TBST and then probed with primary antibodies diluted 1:10,000 in 1× PBS with 1.25% BSA: mouse anti-mCherry (Invitrogen, cat # MA5-32977) and mouse anti-GAPDH (Invitrogen, cat # MA5-15738). HRP-conjugated goat anti-mouse IgG (Invitrogen, cat # A28177) was used as the secondary antibody. Blots were developed using ECL start western blotting detection reagent (Cytiva), and chemiluminescence was detected using Bio-Rad ChemiDoc XRS+ Imaging System.

MG1655 *E. coli* cells were transformed with plasmids expressing GFP, mYpet, and mYpet-MreBCD. Overnight cultures were diluted to 1:100 in LB containing chloramphenicol grown for 3 h and induced with 0.4% arabinose for 3 h. For estimating the native expression levels, the NO50 strain (MreB-msf-GFP^SW^), which expresses GFP-tagged MreB from the native promoter, was used. After induction, equal volumes of samples were collected, washed, and prepared for analysis. Protein samples were separated on a 12% SDS-PAGE gel and transferred to a 0.45 µm nitrocellulose membrane (Cytiva) using the Bio-Rad Mini-PROTEAN Tetra Vertical Electrophoresis System in 1× Tris/glycine buffer. The membranes were blocked with 5% BSA in TBST and then probed with primary antibodies diluted 1:10,000 in 1× PBS with 1.25% mouse anti-GFP (Invitrogen, cat # MA5-15256) and BSA mouse anti-GAPDH (Invitrogen, cat # MA5-15738). Anti-GFP antibody detected mYpet fluorescence protein as well. HRP-conjugated goat anti-mouse IgG (Invitrogen, cat # A28177) was used as the secondary antibody. Blots were developed using ECL start western blotting detection reagent (Cytiva), and chemiluminescence was detected using Bio-Rad ChemiDoc XRS+ Imaging System. Western blot results were quantified, as previously described (42), using Fiji (ImageJ) software, and the graph was plotted using GraphPad Prism.

### Structure prediction

The amino acid sequence of KilR from *E. coli* K12 MG1655 was retrieved from UniProtKB (accession number: P38393). AlphaFold 3 was used to predict the KilR protein structure using default parameters (https://alphafoldserver.com/fold/6fa1f4f62ae1e06b) (23). The resulting three-dimensional structure was visualized and analyzed with PyMol (The PyMOL Molecular Graphics System, Version 3.0 Schrödinger, LLC). Truncated protein structures were generated using PyMOL to explore the structural variations. Additionally, the unstructured regions of KilR were predicted using IUPred2A (https://iupred2a.elte.hu/), employing the IUPred2 short disorder option (24).

### Fluorescence microscopy

Fluorescence microscopy was carried out as described previously (32). Overnight cultures containing appropriate plasmids were grown in LB at 30°C and supplemented with appropriate antibiotics, then subcultured at 1:5 dilutions in fresh LB medium. Cultures were induced with the specified concentration of arabinose and continued to grow for 4 h with slow agitation. Both induced and uninduced samples were collected after 4 h and prepared for microscopy. This standardized condition was used to visualize morphological changes upon expression of KilR or its truncated variants (Fig. 1C and 3C) to assess the effect of mCherry tagging on KilR morphology (see Fig. S4B at https://doi.org/10.5281/zenodo.15803833), study KilR localization using pBAD-mCherry-KilR (Fig. 5A), and visualize MreB-RFP^SW^ and ZapA-GFP in strain SUT106 co-expressing KilR or its variants (Fig. 4).

To assess the morphological effects of KilR from a previously reported plasmid (11), overnight cultures containing the appropriate plasmids were grown in LB at 30°C and supplemented with the appropriate antibiotics. These cultures were then subcultured at a 1:50 dilution in fresh LB medium and grown at 30°C for 2 h with slow agitation. Cultures were induced with the specified concentration of IPTG and continued to grow for an additional 1 h with slow agitation. Both induced and uninduced samples were collected and prepared for microscopy.

To compare the effects of λ Kil and KilR on *E. coli* cell morphology and MreB-msfGFP^SW^ localization, overnight cultures containing the appropriate plasmids were grown in LB at 30°C and supplemented with the appropriate antibiotics. These cultures were then subcultured at a 1:50 dilution in fresh LB medium and grown at 30°C for 2 h with slow agitation. Cultures were induced with the specified concentration of arabinose, shifted to 42°C, and continued to grow for an additional 2 h with slow agitation. Both induced and uninduced samples were collected and prepared for microscopy.

For visualizing mCherry, mCherry-KilR, or KilR together with mYpet-MreBCD, cells containing specified plasmids were grown in LB at 37°C and supplemented with the appropriate antibiotic. The overnight cultures were subcultured at a 1:50 dilution in fresh LB medium and grown at 30°C for 2 h with shaking. Cultures were induced with 0.4% arabinose and continued to grow with slow agitation for 3 h. Samples were collected and prepared for microscopy

To image the localization of MreB and FtsZ under oxidative stress, NO50 and its *ΔkilR* derivative, both containing MreB-msfGFP^SW^ from the native chromosome loci, were transformed with the FtsZ-mCherry plasmid. Overnight cultures were grown in LB at 37°C and supplemented with appropriate antibiotics. Cells were subcultured at a 1:50 dilution in fresh LB medium, then allowed to grow at 30°C for 2 h. H_2_O_2_ concentrations of 0, 3, 5, and 7 mM were added for 1 h, and cells were allowed to grow at 30°C without agitation. Cells with mislocalized FtsZ and mislocalized MreB were identified from each field of view manually with the support of NIS Elements software. Mislocalized FtsZ refers to cells in which the Z-ring is absent, and FtsZ-mCherry signal appeared diffuse/disrupted. Mislocalized MreB refers to cells with aberrant clusters, which are mostly mislocalized to the cell poles and cells with diffuse MreB-msfGFP^SW^ signal. Final data were analyzed and represented as a bar graph using GraphPad Prism. All samples for microscopy were washed with 1× PBS. All processed samples were spotted on 0.8% agarose pads prepared with 1× PBS and visualized and photographed using a Nikon Eclipse Ti2-E equipped with a 100× CFI Plan Apochromat Oil objective and a DSQi-2 monochrome camera.

## Data Availability

Supplemental material is available at https://doi.org/10.5281/zenodo.15803833.

## References

[1] Casjens S. 2003. Prophages and bacterial genomics: what have we learned so far? Mol Microbiol 49:277–300. doi:10.1046/j.1365-2958.2003.03580.x 12886937

[2] Wang X, Kim Y, Ma Q, Hong SH, Pokusaeva K, Sturino JM, Wood TK. 2010. Cryptic prophages help bacteria cope with adverse environments. Nat Commun 1:147. doi:10.1038/ncomms1146 21266997 PMC3105296

[3] Kaiser K, Murray NE. 1979. Physical characterisation of the “Rac prophage” in E. coli K12. Mol Gen Genet 175:159–174. doi:10.1007/BF00425532 390313

[4] Low B. 1973. Restoration by the rac locus of recombinant forming ability in recB - and recC - merozygotes of Escherichia coli K-12. Mol Gen Genet 122:119–130. doi:10.1007/BF00435185 4573863

[5] Hong SH, Wang X, Wood TK. 2010. Controlling biofilm formation, prophage excision and cell death by rewiring global regulator H-NS of Escherichia coli. Microb Biotechnol 3:344–356. doi:10.1111/j.1751-7915.2010.00164.x 21255333 PMC3158429

[6] Liu X, Li Y, Guo Y, Zeng Z, Li B, Wood TK, Cai X, Wang X. 2015. Physiological function of rac prophage during biofilm formation and regulation of rac excision in Escherichia coli K-12. Sci Rep 5:16074. doi:10.1038/srep16074 26530864 PMC4632033

[7] Conter A, Bouché JP, Dassain M. 1996. Identification of a new inhibitor of essential division gene ftsZ as the kil gene of defective prophage Rac. J Bacteriol 178:5100–5104. doi:10.1128/jb.178.17.5100-5104.1996 8752325 PMC178304

[8] Burke C, Liu M, Britton W, Triccas JA, Thomas T, Smith AL, Allen S, Salomon R, Harry E. 2013. Harnessing single cell sorting to identify cell division genes and regulators in bacteria. PLoS One 8:e60964. doi:10.1371/journal.pone.0060964 23565292 PMC3614548

[9] Cardinale CJ, Washburn RS, Tadigotla VR, Brown LM, Gottesman ME, Nudler E. 2008. Termination factor Rho and its cofactors NusA and NusG silence foreign DNA in E. coli. Science 320:935–938. doi:10.1126/science.1152763 18487194 PMC4059013

[10] Krishnamurthi R, Ghosh S, Khedkar S, Seshasayee ASN. 2017. Repression of YdaS toxin Is mediated by transcriptional repressor RacR in the Cryptic rac prophage of Escherichia coli K-12. mSphere 2:10–1128. doi:10.1128/mSphere.00392-17 PMC5700373 29205228

[11] Barshishat S, Elgrably-Weiss M, Edelstein J, Georg J, Govindarajan S, Haviv M, Wright PR, Hess WR, Altuvia S. 2018. OxyS small RNA induces cell cycle arrest to allow DNA damage repair. EMBO J 37:413–426. doi:10.15252/embj.201797651 29237698 PMC5793797

[12] Altuvia S, Weinstein-Fischer D, Zhang A, Postow L, Storz G. 1997. A small, stable RNA induced by oxidative stress: role as a pleiotropic regulator and antimutator. Cell 90:43–53. doi:10.1016/s0092-8674(00)80312-8 9230301

[13] Elgrably-Weiss M, Hussain F, Georg J, Shraiteh B, Altuvia S. 2024. Balanced cell division is secured by two different regulatory sites in OxyS RNA. RNA 30:124–135. doi:10.1261/rna.079836.123 38071477 PMC10798246

[14] Masuda H, Tan Q, Awano N, Yamaguchi Y, Inouye M. 2012. A novel membrane-bound toxin for cell division, CptA (YgfX), inhibits polymerization of cytoskeleton proteins, FtsZ and MreB, in Escherichia coli. FEMS Microbiol Lett 328:174–181. doi:10.1111/j.1574-6968.2012.02496.x 22239607 PMC3334289

[15] Tan Q, Awano N, Inouye M. 2011. YeeV is an Escherichia coli toxin that inhibits cell division by targeting the cytoskeleton proteins, FtsZ and MreB. Mol Microbiol 79:109–118. doi:10.1111/j.1365-2958.2010.07433.x 21166897 PMC3021753

[16] Heller DM, Tavag M, Hochschild A. 2017. CbtA toxin of Escherichia coli inhibits cell division and cell elongation via direct and independent interactions with FtsZ and MreB. PLoS Genet 13:e1007007. doi:10.1371/journal.pgen.1007007 28931012 PMC5624674

[17] Wen Z, Wang P, Sun C, Guo Y, Wang X. 2017. Interaction of Type IV Toxin/Antitoxin systems in cryptic prophages of Escherichia coli K-12. Toxins (Basel) 9:77. doi:10.3390/toxins9030077 28257056 PMC5371832

[18] Greer H. 1975. The kil gene of bacteriophage lambda. Virology (Auckl) 66:589–604. doi:10.1016/0042-6822(75)90231-7 1098278

[19] Haeusser DP, Hoashi M, Weaver A, Brown N, Pan J, Sawitzke JA, Thomason LC, Court DL, Margolin W. 2014. The Kil peptide of bacteriophage λ blocks Escherichia coli cytokinesis via ZipA-dependent inhibition of FtsZ assembly. PLoS Genet 10:e1004217. doi:10.1371/journal.pgen.1004217 24651041 PMC3961180

[20] Hernández-Rocamora VM, Alfonso C, Margolin W, Zorrilla S, Rivas G. 2015. Evidence that bacteriophage λ kil peptide inhibits bacterial cell division by disrupting FtsZ Protofilaments and sequestering protein subunits. J Biol Chem 290:20325–20335. doi:10.1074/jbc.M115.653329 26124275 PMC4536439

[21] Geissler B, Elraheb D, Margolin W. 2003. A gain-of-function mutation in ftsA bypasses the requirement for the essential cell division gene zipA in Escherichia coli. Proc Natl Acad Sci U S A 100:4197–4202. doi:10.1073/pnas.0635003100 12634424 PMC153070

[22] Young KD. 2008. Why spherical Escherichia coli dies: the inside story. J Bacteriol 190:1497–1498. doi:10.1128/JB.01975-07 18165295 PMC2258677

[23] Abramson J, Adler J, Dunger J, Evans R, Green T, Pritzel A, Ronneberger O, Willmore L, Ballard AJ, Bambrick J, et al.. 2024. Accurate structure prediction of biomolecular interactions with AlphaFold 3. Nature New Biol 630:493–500. doi:10.1038/s41586-024-07487-w PMC11168924 38718835

[24] Erdős G, Dosztányi Z. 2020. Analyzing protein disorder with IUPred2A. CP in Bioinformatics 70:e99. doi:10.1002/cpbi.99 32237272

[25] Weber M, Burgos R, Yus E, Yang J-S, Lluch-Senar M, Serrano L. 2020. Impact of C-terminal amino acid composition on protein expression in bacteria. Mol Syst Biol 16:e9208. doi:10.15252/msb.20199208 32449593 PMC7246954

[26] Lutkenhaus J. 1993. FtsZ ring in bacterial cytokinesis. Mol Microbiol 9:403–409. doi:10.1111/j.1365-2958.1993.tb01701.x 8412689

[27] McQuillen R, Xiao J. 2020. Insights into the structure, function, and dynamics of the bacterial cytokinetic FtsZ-Ring. Annu Rev Biophys 49:309–341. doi:10.1146/annurev-biophys-121219-081703 32092282 PMC8610174

[28] Govindarajan S, Nevo-Dinur K, Amster-Choder O. 2012. Compartmentalization and spatiotemporal organization of macromolecules in bacteria. FEMS Microbiol Rev 36:1005–1022. doi:10.1111/j.1574-6976.2012.00348.x 22775310

[29] Errington J. 2015. Bacterial morphogenesis and the enigmatic MreB helix. Nat Rev Microbiol 13:241–248. doi:10.1038/nrmicro3398 25578957

[30] Bendezú FO, Hale CA, Bernhardt TG, de Boer PAJ. 2009. RodZ (YfgA) is required for proper assembly of the MreB actin cytoskeleton and cell shape in E. coli. EMBO J 28:193–204. doi:10.1038/emboj.2008.264 19078962 PMC2637328

[31] Peters NT, Dinh T, Bernhardt TG. 2011. A fail-safe mechanism in the septal ring assembly pathway generated by the sequential recruitment of cell separation amidases and their activators. J Bacteriol 193:4973–4983. doi:10.1128/JB.00316-11 21764913 PMC3165665

[32] Govindarajan S, Amster-Choder O. 2017. The bacterial Sec system is required for the organization and function of the MreB cytoskeleton. PLoS Genet 13:e1007017. doi:10.1371/journal.pgen.1007017 28945742 PMC5629013

[33] Gueiros-Filho FJ, Losick R. 2002. A widely conserved bacterial cell division protein that promotes assembly of the tubulin-like protein FtsZ. Genes Dev 16:2544–2556. doi:10.1101/gad.1014102 12368265 PMC187447

[34] Galli E, Gerdes K. 2010. Spatial resolution of two bacterial cell division proteins: ZapA recruits ZapB to the inner face of the Z-ring. Mol Microbiol 76:1514–1526. doi:10.1111/j.1365-2958.2010.07183.x 20487275

[35] Kruse T, Bork-Jensen J, Gerdes K. 2005. The morphogenetic MreBCD proteins of Escherichia coli form an essential membrane-bound complex. Mol Microbiol 55:78–89. doi:10.1111/j.1365-2958.2004.04367.x 15612918

[36] Shiomi D, Sakai M, Niki H. 2008. Determination of bacterial rod shape by a novel cytoskeletal membrane protein. EMBO J 27:3081–3091. doi:10.1038/emboj.2008.234 19008860 PMC2599877

[37] Yakhnina AA, Gitai Z. 2012. The small protein MbiA interacts with MreB and modulates cell shape in Caulobacter crescentus*.* Mol Microbiol 85:1090–1104. doi:10.1111/j.1365-2958.2012.08159.x 22804814 PMC4105008

[38] Fenton AK, Gerdes K. 2013. Direct interaction of FtsZ and MreB is required for septum synthesis and cell division in Escherichia coli. EMBO J 32:1953–1965. doi:10.1038/emboj.2013.129 23756461 PMC3708099

[39] Ursell TS, Nguyen J, Monds RD, Colavin A, Billings G, Ouzounov N, Gitai Z, Shaevitz JW, Huang KC. 2014. Rod-like bacterial shape is maintained by feedback between cell curvature and cytoskeletal localization. Proc Natl Acad Sci USA 111:E1025–34. doi:10.1073/pnas.1317174111 24550515 PMC3964057

[40] Govindarajan S, Borges A, Karambelkar S, Bondy-Denomy J. 2022. Distinct subcellular localization of a type I CRISPR complex and the Cas3 nuclease in bacteria. J Bacteriol 204:e0010522. doi:10.1128/jb.00105-22 35389256 PMC9112876

[41] Belitsky M, Avshalom H, Erental A, Yelin I, Kumar S, London N, Sperber M, Schueler-Furman O, Engelberg-Kulka H. 2011. The Escherichia coli extracellular death factor EDF induces the endoribonucleolytic activities of the toxins MazF and ChpBK. Mol Cell 41:625–635. doi:10.1016/j.molcel.2011.02.023 21419338

[42] Taylor SC, Posch A. 2014. The design of a quantitative western blot experiment. Biomed Res Int 2014:361590. doi:10.1155/2014/361590 24738055 PMC3971489

